# Usefulness of ultrasonography to confirm hemostasis after femoral artery access in endovascular therapy

**DOI:** 10.1186/s42155-026-00674-y

**Published:** 2026-03-18

**Authors:** Yuki Shima, Risa Bando, Gakuto Bando, Narumi Irie, Kazunori Mushiake, Hiroyuki Tanaka, Mitsuru Abe

**Affiliations:** 1 Department of Cardiovascular Medicine, Kurashiki Central Hospital, 1-1-1 Miwa, Kurashiki, 710-8602 Japan; 2 Department of Clinical Laboratory, Kurashiki Central Hospital, 1-1-1 Miwa, Kurashiki, 710-8602 Japan

**Keywords:** Peripheral artery disease, Endovascular therapy, Hemostasis, Ultrasonography

## Abstract

**Background:**

Femoral artery access is commonly used in endovascular therapy (EVT) for peripheral artery disease (PAD), but puncture site complications remain a significant concern. Although manual compression is widely applied, assessment of hemostasis is often subjective. Routine ultrasonographic confirmation of hemostasis may provide a more objective evaluation, but its clinical impact has not been fully clarified. The purpose of this study is to investigate the association between ultrasonography-confirmed hemostasis and femoral artery access site complications following EVT for PAD.

**Materials and methods:**

This single-center retrospective cohort study analysed 1643 femoral artery access sites in patients undergoing EVT for PAD between January 2018 and August 2025. Until December 2019, hemostasis was assessed based on clinical judgement alone, whereas from January 2020 onward, routine ultrasonographic confirmation of hemostasis was implemented as an institutional protocol. Access sites were categorized into an ultrasonography group (*n* = 1185) and a non-ultrasonography group (*n* = 458). A 1:1 propensity score matching analysis yielded 429 matched access sites in each group. The primary endpoint was access site bleeding complications, defined as persistent or recurrent bleeding, hematoma formation, or pseudoaneurysm development during or after compression with a pressure bandage.

**Results:**

In the matched cohort, ultrasonography-confirmed hemostasis was associated with a significantly lower incidence of bleeding complications when 6 Fr sheaths were used (3.5% vs. 7.7%, *p* = 0.022). The incidence of pseudoaneurysm formation was also lower in the ultrasonography group with 6 Fr sheaths (0.95% vs. 3.5%, *p* = 0.029). No significant differences in bleeding complications were observed between groups when 5 Fr or smaller were used. Within the ultrasonography group, bleeding complication rates did not differ between antegrade and retrograde femoral access approaches.

**Conclusions:**

Routine ultrasonographic confirmation of femoral artery hemostasis was associated with fewer access site bleeding complications after EVT for PAD, particularly when larger (6 Fr) sheaths were used. Ultrasonographic evaluation may represent a simple and effective strategy to enhance procedural safety in femoral artery EVT.

## Introduction

Endovascular therapy (EVT) is widely performed for peripheral artery disease (PAD) presenting with claudication and chronic limb-threatening ischemia [1]. In recent days, radial artery access has become one option for aortoiliac EVT because of its potential to reduce procedural complications [2–4]. However, for femoropopliteal and below-the-knee artery lesions, EVT is generally performed via femoral artery access. In addition, the percutaneous puncture of the femoral artery is widely used across various medical fields, including vascular surgery, cardiology, interventional radiology, and neuroradiology [5]. Femoral artery puncture site complications are one of the significant complications that can occur following interventions [6–8]. Typically, manual compression is applied, and once the operator determines hemostasis has been achieved, each patient was given a pressure bandage. However, this determination is subjective and not an objective assessment. The puncture site complications occur at a certain rate, and the methods of hemostasis also vary among hospitals. Therefore, performing an ultrasonography assessment of puncture site allows for objective confirmation of whether hemostasis has been achieved. Ultrasonography evaluation is quick and minimally invasive for patients.

The purpose of this study is to evaluate the effectiveness and safety of hemostasis at femoral artery access site as assessed by ultrasonography following EVT.

## Methods

### Study population and design

This study was conducted as a single-center, retrospective cohort study designed to evaluate the impact of ultrasonography-confirmed hemostasis on femoral artery access site complications following EVT for PAD. We reviewed consecutive patients with PAD who underwent EVT via femoral access at our institution between January 2018 to August 2025. Clinical, procedural, and imaging data were obtained from electronic medical records and procedural reports. Informed consent was given by all patients for the procedure and subsequent data collection and analysis for research purpose, and the study was approved by the institutional ethics committee.

### Inclusion and exclusion criteria

Femoral artery access sites were included if EVT was performed using a femoral approach. Access sites were excluded if non-femoral access was used, bilateral femoral access was performed during the same procedure, or if a sheath size of 7 Fr or larger was used.

### Group assignment

Femoral access sites were categorized according to the institutional hemostasis protocol in place at the time of the procedure. Until December 2019, hemostasis was assessed based on clinical judgement alone without routine ultrasonographic evaluation. From January 2020 onward, ultrasonography was routinely performed in all cases to confirm hemostasis after manual compression and before application of pressure bandage.

### Procedural protocol

**Fig. 1:**
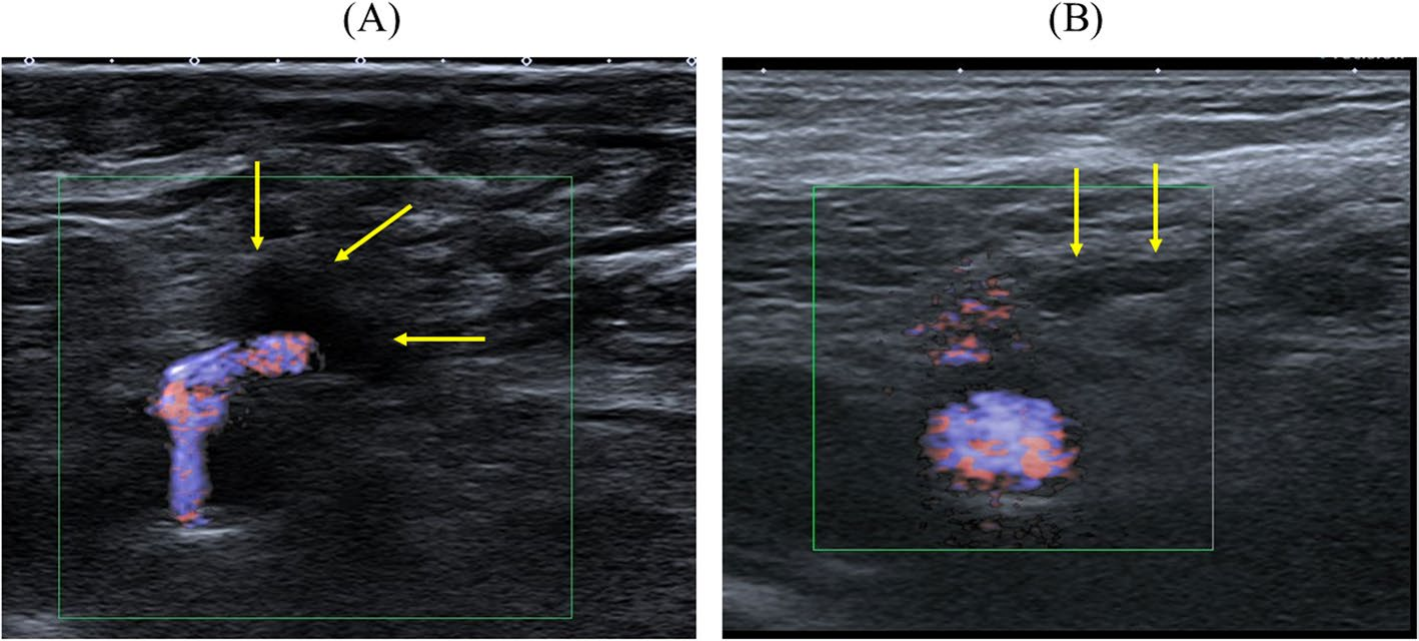
The ultrasonography images of the access site (**A**) inflow of blood from the puncture site into the hematoma (**B**) no blood flow into the hematoma was observed

Endovascular therapy was performed via an ipsilateral antegrade approach or crossover approach from the common femoral artery. The anticoagulant employed was unfractionated heparin. At the end of the EVT, a vascular closure device (Exoseal; Cordis Corporation, Bridgewater, NJ, USA) was applied in most cases, followed by a short manual compression of about 10 min. Compression was maintained until the operator determined hemostasis had been achieved. In the ultrasonography group, duplex ultrasonography was performed by the operator or a trained sonographer to confirm the absence of active bleeding, hematoma formation, or pseudoaneurysm prior to application of pressure bandage. Ultrasonography was performed immediately after manual compression and before application of the pressure bandage. The examination was performed using a high-frequency linear probe and focused on the puncture site to confirm absence of active bleeding, hematoma, or pseudoaneurysm (Fig. 1). The assessment typically required less than 1 min. In the non-ultrasonography group, compression with pressure bandage was applied without ultrasonographic assessment. According to institutional protocol, the pressure bandage was maintained for approximately 3 h in both groups. An Exoseal was routinely used for femoral artery closure at our institution throughout the study period. Although Exoseal was used in most procedures (estimated > 95%), the exact number of cases could not be fully retrieved due to incomplete procedural documentation.

The antiplatelet drug administered was aspirin (100 mg/day) with clopidogrel (75 mg/day) and/or cilostazol (100 or 200 mg/day), and its duration was dependent on the patient’s background, lesion characteristics, and intervention results. At least one antiplatelet drug was maintained after EVT for all patients.

### Study endpoints and definitions

The rate of bleeding complications was analyzed as endpoints. The bleeding complications were defined as (1) persistent or recurrent bleeding at the femoral access site that occurred during or after pressure bandage compression and required additional manual compression or prolonged compression time. (2) Oozing or hematoma formation at the access site judged by the operator or sonographer as indicating inadequate hemostasis. (3) Development of a pseudoaneurysm, confirmed by duplex ultrasonography after the procedure. In both group, bleeding complications were identified during index hospitalization based on clinical assessment and, when clinically indicated, duplex ultrasonography. In the ultrasonography group, the immediate post-compression examination was part of the protocol, whereas in the non-ultrasonography group, ultrasonography was performed only if clinically suspected. All patients were clinically observed during the index hospitalization, which was typically 2 days in the absence of complications.

### Statistical analysis

Categorical variables were compared using the chi-square test. Continuous variables are expressed as mean ± standard deviation and were compared using Student’s t-test or the Wilcoxon rank-sum test based on the distributions. The Kolmogorov–Smirnov test was used to test the normality of the distribution of all quantitative variables. To reduce confounding related to baseline differences, we performed 1:1 propensity score-matching analysis. Propensity scores were estimated using a logistic regression model in which the treatment assignment (ultrasonography vs non-ultrasonography) was regressed on clinically relevant baseline variables, including age, sex, body mass index, diabetes mellitus, hypertension, smoking history, hemodialysis, sheath size, and use of aspirin, P2Y12 inhibitor, cilostazol, warfarin and direct oral anticoagulant. Statistical analysis was performed using JMP (version 18.0, SAS Institute, Cary, NC, USA).

## Results

### Study population and flow

**Fig. 2:**
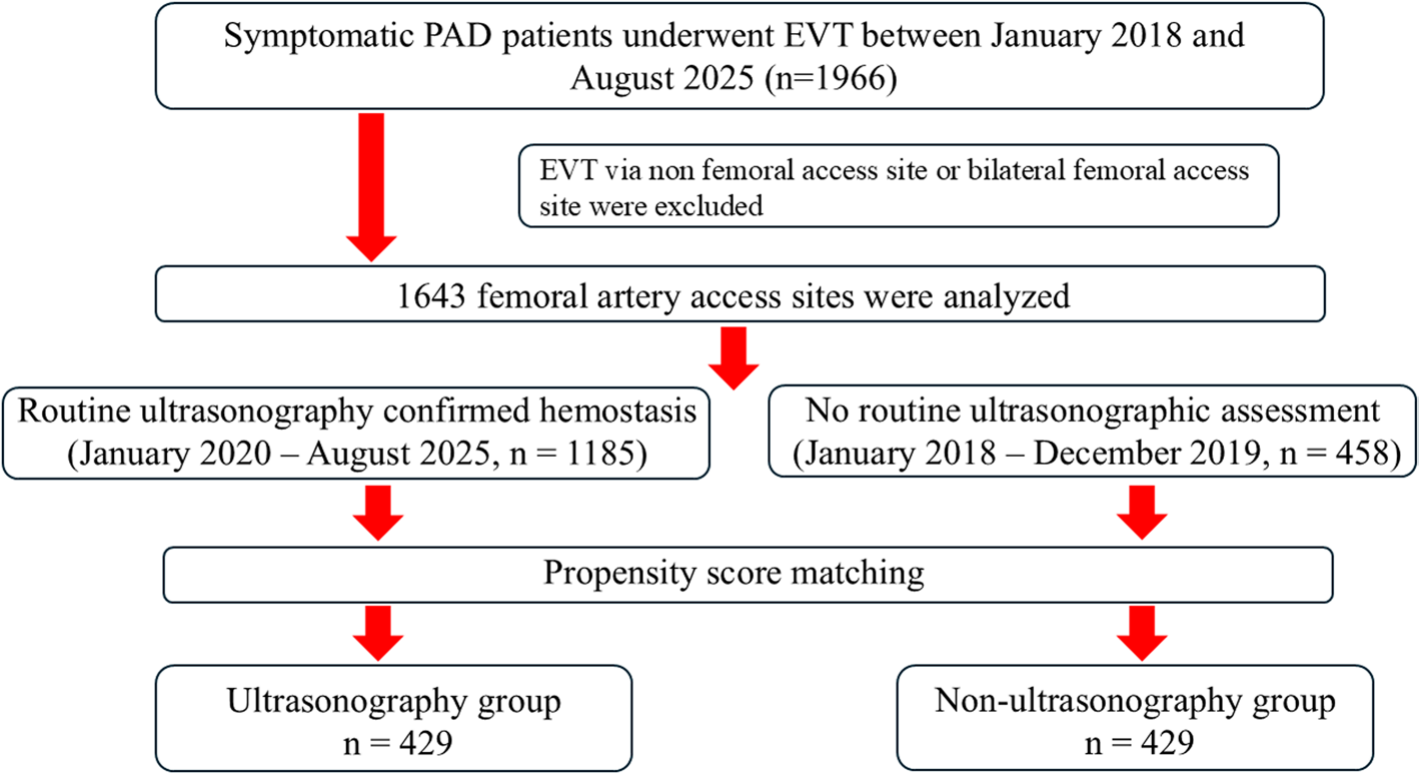
Study flow chart. PAD = peripheral artery disease, EVT = endovascular therapy. Femoral artery access sites were categorized according to the institutional hemostasis protocol. From January 2018 to December 2019, hemostasis was assessed without routine ultrasonography, whereas from January 2020 onward, ultrasonography was routinely performed to confirm hemostasis after manual compression. After propensity score matching, 429 access sites were included in each group

A total of 1966 patients underwent EVT for PAD during the study period. After applying the exclusion criteria, 1643 femoral artery access sites were included in the analysis. According to the institutional hemostasis protocol, 458 access sites were treated without routine ultrasonographic assessment between January 2018 and December 2019, whereas 1185 access sites underwent routine ultrasonography-confirmed hemostasis from January 2020 to August 2025 (Fig. 2). After propensity score matching, 429 access sites were included in each group.

### Baseline characteristics

The baseline characteristics before and after propensity score matching are summarized in Table 1. In the matched cohort, there were no significant differences between the ultrasonography and non-ultrasonography groups in terms of age, sex, body mass index, comorbidities, antithrombotic therapy, sheath size, or access approach. The year of the procedure was balanced between groups after propensity score matching.

**Table 1:** The baseline characteristics of the study population

	Overall population			Matched population			
	Ultrasonography (+)	Ultrasonography (−)	*P* value	Ultrasonography (+)	Ultrasonography (−)	*P* value	SD (%)
Patients, *n*	1185	458		429	429		
Age, years	75.7 ± 9.3	74.2 ± 9.4	0.0036	74.6 ± 9.9	74.3 ± 9.2	0.6	3.1
Male, *n* (%)	745 (62.9)	292 (63.7)	0.74	270 (62.9)	273 (63.7)	0.83	1.4
BMI	21.7 ± 3.7	21.8 ± 3.3	0.74	21.8 ± 3.5	21.8 ± 3.3	0.96	0.3
Hypertension, *n* (%)	949 (80.1)	353 (77.1)	0.18	341 (79.5)	337 (78.6)	0.34	1.8
Hyperlipidemia, *n* (%)	697 (58.8)	248 (54.3)	0.094	244 (56.9)	236 (55.0)	0.58	3.1
Diabetes mellitus, *n* (%)	727 (61.4)	282 (61.8)	0.85	283 (65.9)	264 (61.5)	0.17	7.4
Smoking history, *n* (%)	664 (56.0)	317 (69.2)	< 0.001	21 (65.5)	293 (68.3)	0.22	4.9
Hemodialysis, *n* (%)	403 (34.0)	170 (37.1)	0.24	158 (36.8)	157 (36.9)	0.94	0.16
Aspirin, *n* (%)	813 (68.6)	347 (75.8)	0.0043	333 (77.6)	328 (76.5)	0.68	2.1
P2Y12 inhibitor, *n* (%)	913 (77.1)	301 (65.7)	< 0.001	298 (69.5)	300 (69.9)	0.88	0.71
Cilostazol, *n* (%)	112 (9.5)	60 (13.1)	0.03	56 (13.1)	57 (13.3)	0.92	0.48
Direct oral anticoagulant, *n* (%)	117 (9.9)	34 7.4)	0.12	26 (6.1)	31 (7.2)	0.49	3.6
Warfarin, n (%)	90 (7.6)	37 (8.1)	0.74	32 (7.5)	34 (7.9)	0.8	1.2
DAPT + anticoagulant, n (%)	25 (2.1)	16 (3.5)	0.11	13 (3.0)	15 (3.5)	0.7	2.3
DAPT, *n* (%)	691 (58.3)	258 (56.3)	0.47	264 (61.5)	257 (59.9)	0.62	2.7
SAPT + anticoagulant, *n* (%)	134 (11.3)	43 (9.4)	0.26	35 (8.2)	42 (9.8)	0.4	4.5
SAPT, *n* (%)	274 (23.1)	118 (25.8)	0.26	99 (23.1)	100 (23.3)	0.94	0.39
Anticoagulant alone, *n* (%)	54 (4.6)	12 (2.6)	0.073	11 (2.6)	10 (2.3)	0.83	1.6
Sheath size							
4.5 + 5 Fr,* n* (%)	559 (47.2)	120 (26.2)	< 0.001	114 (26.6)	117 (27.3)	0.82	1.3
6 Fr, n (%)	626 (52.8)	338 (73.8)	< 0.001	315 (73.4)	312 (72.7)	0.82	1.3
Antegrade access, *n* (%)	810 (66.9)	467 (67.8)	0.71	272 (63.4)	273 (63.6)	0.94	0.34

Values are mean ± SD or numbers (%), unless otherwise specified

*BMI *body mass index, *DAPT *dual anti-platelet therapy, *SAPT *single anti-platelet therapy

### Primary endpoint: bleeding complications

**Fig. 3:**
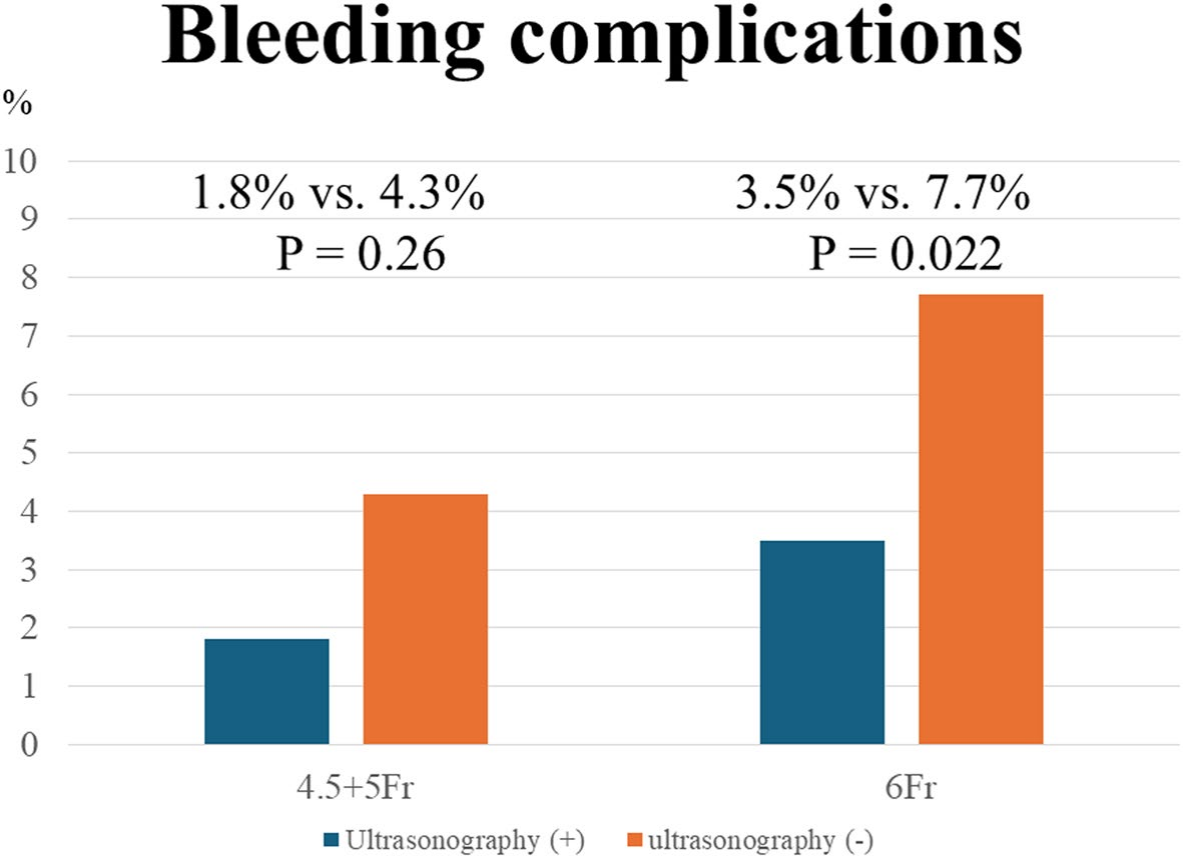
Rates of bleeding complication

Figure 3 shows the bleeding complications among two groups. With 6Fr sheaths, the ultrasonography group had significantly fewer bleeding complications (3.5% vs. 7.7%, *p* = 0.022). On the other hand, with 4.5 and 5Fr sheath, the bleeding complications were no significant difference between two groups (1.8% vs. 4.3%, *p* = 0.26).

### Types of bleeding complications

The types of bleeding complications are summarized in Table 2. Among access sites using 6 Fr sheaths, the incidence of pseudoaneurysm formation was significantly lower in the ultrasonography group than non-ultrasonography group (0.95% vs. 3.5%, *p* = 0.029). Among the cases where the pseudoaneurysm formed, only one treated by the local injection of thrombin and the subsequent application of a pressure bandage in the non-ultrasonography group, while the others achieved hemostasis through manual compression.

**Table 2:** The bleeding complications

	Ultrasonography (+)	Ultrasonography (−)	*P* value
Bleeding complications, *n* (%)			
Overall	13 (3.0)	29 (6.8)	0.011
4.5 + 5Fr	2 (1.8)	5 (4.3)	0.26
6Fr	11 (3.5)	24 (7.7)	0.022
Bleeding, *n* (%)			
Overall	13 (3.0)	29 (6.8)	0.011
4.5 + 5Fr	2 (1.8)	5 (4.3)	0.26
6Fr	11 (3.5)	24 (7.7)	0.022
Pseudoaneurysm, (%)			
Overall	5 (1.2)	15 (3.5)	0.023
4.5 + 5Fr	1 (0.88)	4 (3.4)	0.18
6Fr	3 (0.95)	11 (3.5)	0.029

Values are numbers (%)

### Subgroup analysis: access approach

Among access sites in the ultrasonography group using 6 Fr sheath, 266 procedures were performed via an ipsilateral antegrade approach and 360 via a retrograde approach. The incidence of bleeding complications did not differ significantly between the antegrade and retrograde approaches (3.4% vs. 3.1%, *p* = 0.82) (Table 3).

**Table 3:** The bleeding complications according to access site

	Antegrade	Retrograde	*P* value
Bleeding complications, *n* (%)	9 (3.4)	11 (3.1)	0.82
Bleeding, *n* (%)	9 (3.4)	11 (3.1)	0.82
Pseudoaneurysm, (%)	4 (1.5)	6 (1.7)	0.87

Values are numbers (%)

### Other clinical outcomes

No access site-related ischemic complications, limb loss, or procedure-related mortality were observed during the index hospitalization in either group.

## Discussion

The present study demonstrated that routine ultrasonography-confirmed hemostasis after femoral artery access was associated with a lower incidence of access site bleeding complications following EVT for PAD, particularly when larger (6 Fr) sheaths were used. This benefit was observed after the introduction of an institution-wide hemostasis protocol incorporating ultrasonographic assessment.

Femoral artery access remains the standard approach for EVT for PAD. Although vascular closure device and manual compression are widely used, confirmation of complete hemostasis is often based on subjective clinical judgement. Ultrasonography allows direct visualization of the puncture site and surrounding tissue, enabling objective confirmation of adequate hemostasis before pressure bandage application. The ultrasonographic assessment was performed as a focused evaluation of the puncture site using a linear probe and typically required less than 1 min. No formal learning curve was observed, as the examination was limited to confirmation of absence of active bleeding or pseudoaneurysm. Therefore, the additional procedural burden was minimal. The present findings suggest that this simple additional step may improve procedural safety in routine clinical practice.

In this study, the reduction in bleeding complication was most pronounced in procedures using 6 Fr sheaths, whereas no significant difference was observed with smaller sheath sizes. Some reports demonstrated an incidence of vascular access site–related complication rate that ranged from 0.6 to 9% [8–10]. Compared to these studies, the rate of bleeding complications in this study appears to be somewhat lower, particularly in the group evaluated by ultrasonography. Among pseudoaneurysm cases, only one required thrombin injection, and none required surgical repair, indicating that early ultrasonographic detection may allow timely and minimally invasive management. According to previous studies, bleeding complications are associated with older age, sheath size, anticoagulation and obesity in transfemoral catheterization [11, 12]. In this study as well, the complication rate was higher with 6 Fr sheaths than with sheaths of 5 Fr or smaller. Thus, the larger the sheath is used, the more secure hemostasis is required. Obesity patients are also considered to have a higher risk of puncture site complications because visual confirmation of hemostasis is difficult. However, ultrasonography examination enables objective evaluation of hemostasis. Confirming hemostasis with ultrasonography significantly reduced puncture site complications, suggesting it may be an effective method. This strategy can be easily implemented in daily interventional practice without additional devices or substantial cost.

EVT via the femoral artery access requires choosing between ipsilateral antegrade approach or retrograde approach. Although the effect of antegrade approach on access site complications has been reported, with conflicting results about whether antegrade approach increases access site complications [13–15]. In this study, both groups showed a slightly higher proportion of antegrade approach at about 60%. Recently, Ramirez et al. reported that antegrade approach is associated with increased access site complications in large scale data [16]. The antegrade approach is often more difficult and requires experience, but this approach remains an effective method alongside the traditional retrograde approach. In the present study, no significant difference in bleeding complications was observed between antegrade and retrograde approaches within the ultrasonography group, suggesting that routine ultrasonographic assessment may help standardize hemostasis regardless of access strategy.

## Limitations

This study has four major limitations: First, this was a single-center, retrospective study, and residual confounding cannot be completely excluded despite propensity score matching. As this study compares two time periods before and after implementation of routine ultrasonography, improvements in operator experience or procedural technique over time cannot be fully excluded. Although propensity score matching was performed, residual confounding due to temporal trends remains possible. Second, the study design reflected a before-and-after comparison following the implementation of a routine ultrasonography-based hemostasis protocol. Therefore, unmeasured temporal factors may have influenced outcomes. Third, although Exoseal closure devices were routinely used in more than 95% of cases at our institution, the exact rate of device use in each group could not be fully ascertained due to incomplete procedural documentation. Therefore, residual confounding related to closure device use cannot be completely excluded. However, given the consistent institutional practice throughout the study period, substantial imbalance between the two groups is unlikely. Fourth, the exact duration of manual compression was not systematically recorded in our database and therefore could not be compared between two groups. Finally, the study was not powered to detect differences in rare but severe access site complications.

## Conclusions

Routine ultrasonographic confirmation of femoral artery hemostasis was associated with fewer access site bleeding complications after EVT for PAD, particularly when larger (6 Fr) sheaths were used. Ultrasonographic evaluation may represent a simple and effective strategy to enhance procedural safety in femoral artery EVT.

## Data Availability

The datasets used and/or analyzed during the current study are available from the corresponding author upon reasonable request.

## Abbreviations

EVT: Endovascular therapy
PAD: Peripheral artery disease

## References

[1] Aboyans V, Ricco JB, Bartelink MEL, Björck M, Brodmann M, Cohnert T, et al. 2017 ESC guidelines on the diagnosis and treatment of peripheral arterial diseases, in collaboration with the European society for vascular surgery (ESVS): Document covering atherosclerotic disease of extracranial carotid and vertebral, mesenteric, renal, upper and lower extremity arteries Endorsed by: the European Stroke Organization (ESO) The Task Force for the Diagnosis and Treatment of Peripheral Arterial Diseases of the European Society of Cardiology (ESC) and of the European Society for Vascular Surgery (ESVS). Eur Heart J. 2018;39(9):763–816. 28886620 10.1093/eurheartj/ehx095

[2] Iida O, Takahara M, Fujihara M, Higashino N, Hayakawa N, Horie K, et al. Clinical outcomes of transradial vs nontransradial aortoiliac endovascular therapy. JACC Cardiovasc Interv. 2024;17(16):1891–901. 39197987 10.1016/j.jcin.2024.06.002

[3] Meertens MM, Ng E, Loh SEK, Samuel M, Mees BME, Choong AMTL. Transradial approach for aortoiliac and femoropopliteal interventions; a systematic review and meta-analysis. J Endovasc Ther. 2018;25:599–607. 30086665 10.1177/1526602818792854 PMC6136071

[4] Ruzsa Z, Bellavics R, Nemes B, Hüttl A, Nyerges A, Sótonyi P, et al. Combined transradial and transpedal approach for femoral artery interventions. JACC: Cardiovasc Interv. 2018;11:1062–71. 10.1016/j.jcin.2018.03.038 29880100

[5] Uhl C, Hatzl J, Meisenbacher K, Zimmer L, Hartmann N, Böckler D. Mixed-reality-assisted puncture of the common femoral artery in a phantom model. J Imaging. 2022;8(2):47. 35200749 10.3390/jimaging8020047 PMC8874567

[6] Gabriel M, Pawlaczyk K, Waliszewski K, Krasiński Z, Majewski W. Location of femoral artery puncture site and the risk of postcatheterization pseudoaneurysm formation. Int J Cardiol. 2007;120(2):167–71. 17250906 10.1016/j.ijcard.2006.09.018

[7] Thalhammer C, Kirchherr AS, Uhlich F, Waigand J, Gross SM. Postcatheterization pseudoaneurysms and arteriovenous fistulas: repair with percutaneous implantation of endovascular covered stents. Radiology. 2000;214(1):127–31. 10644111 10.1148/radiology.214.1.r00ja04127

[8] Cacuci AC, Krankenberg H, Ingwersen M, Gayed M, Stein SD, Kretzschmar D, et al. Access site complications of peripheral endovascular procedures: a large, prospective registry on predictors and consequences. J Endovasc Ther. 2021;28(5):746–54. 34137662 10.1177/15266028211025044

[9] Kolluri R, Fowler B, Nandish S. Vascular access complications: diagnosis and management. Curr Treat Options Cardiovasc Med. 2013;15:173–87. 23378180 10.1007/s11936-013-0227-8

[10] Hackl G, Gary T, Belaj K, Hafner F, Rief P, Deutschmann H, et al. Exoseal for puncture site closure after antegrade procedures in peripheral arterial disease patients. Diagn Interv Radiol. 2014;20:426–31. 25010369 10.5152/dir.2014.14002 PMC4463333

[11] Benamer H, Louvard Y, Sanmartin M, Valsecchi O, Hildick-Smith D, Garot P, et al. A multicentre comparison of transradial and transfemoral approaches for coronary angiography and PTCA in obese patients: the TROP registry. EuroIntervention. 2007;3:327–32. 19737713 10.4244/eijv3i3a60

[12] Doyle BJ, Ting HH, Bell MR, Lennon RJ, Mathew V, Singh M, et al. Major femoral bleeding complications after percutaneous coronary intervention: incidence, predictors, and impact on long-term survival among 17,901 patients treated at the Mayo Clinic from 1994 to 2005. JACC Cardiovasc Interv. 2008;1:202–9. 19463301 10.1016/j.jcin.2007.12.006

[13] Cragg J, Lowry D, Hopkins J, Parker D, Kay M, Duddy M, et al. Safety and outcomes of ipsilateral antegrade angioplasty for femoropopliteal disease. Vasc Endovascular Surg. 2018;52:93–7. 29237359 10.1177/1538574417739762

[14] Wheatley BJ, Mansour MA, Grossman PM, Munir K, Cali RF, Gorsuch JM, et al. Complication rates for percutaneous lower extremity arterial antegrade access. Arch Surg. 2011;146:432–5. 21502451 10.1001/archsurg.2011.47

[15] Biondi-Zoccai GG, Agostoni P, Sangiorgi G, Dalla Paola L, Armano F, Nicolini S, et al. Mastering the antegrade femoral artery access in patients with symptomatic lower limb ischemia: learning curve, complications, and technical tips and tricks. Catheter Cardiovasc Interv. 2006;68:835–42. 17086526 10.1002/ccd.20930

[16] Ramirez JL, Smith EJT, Zarkowsky DS, Lopez J, Hicks CW, Schneider PA, et al. Closure device use for common femoral artery antegrade access is higher risk than retrograde access. Ann Vasc Surg. 2021;76:49–58. 33838236 10.1016/j.avsg.2021.03.009 PMC9869430

